# Effects of Vertical Spatial Overlap on Phytoplankton Diversity under Experimentally Altered Lake Stratification Regimes

**DOI:** 10.3390/microorganisms9122447

**Published:** 2021-11-27

**Authors:** Philippe Le Noac’h, Vincent Ouellet Jobin, Beatrix E. Beisner

**Affiliations:** Department of Biological Sciences, University of Québec at Montréal and Interuniversity Research Group in Limnology/Groupe de Recherche Interuniversitaire en Limnologie (GRIL), Succ. Centre-Ville, Montréal, QC 8888, Canada; vincent.oj@gmail.com (V.O.J.); beisner.beatrix@uqam.ca (B.E.B.)

**Keywords:** diversity, composition, functional traits, competition, spatial ecology

## Abstract

In phytoplankton communities, competitive exclusion might occur when functionally similar species are impeded from regulating their positions along light and nutrient gradients to reduce niche overlap. Greater spatial overlap (*SO*) between species due to water column mixing could thus promote competitive exclusion, reducing community taxonomic diversity. However, greater *SO* could also promote coexistence of functionally different taxa. Using data from a whole-lake experiment, we investigated the effects of *SO* and other relevant environmental factors on phytoplankton diversity across the water columns of lake basins with different thermocline manipulations. We estimated *SO* using an in situ fluorometer, and overall community diversity microscopically. Using structured equation models, we estimated directional relationships between phytoplankton diversity, *SO*, the lake physical structure and the zooplankton community. No significant effect of *SO* on phytoplankton taxonomic diversity was observed, but higher *SO* was associated with greater functional diversity. Change in lake physical structure and in the zooplankton community also affected diversity, with a negative response to increased top-down interactions. Overall, despite the fact that the alteration of water column stratification structure and top-down interactions were stronger drivers of phytoplankton diversity in our system, some effect of spatial overlap on the outcome of inferred competitive interactions were observable.

## 1. Introduction

Spatial segregation along opposing resource gradients constitutes one of the mechanisms allowing multiple species to coexist on a finite number of resources [1,2]. In thermally stratified lakes, two important gradients for phytoplankton growth are represented by light and nutrient concentrations. Light decreases from a lake’s surface, and nutrient concentrations increase at depth, thereby forming opposing gradients of essential resources. In this context, studies have theorized about the importance of phytoplankton segregation in the water column to mitigate interspecific competition [3,4]. Under stratified conditions, species can actively establish at different depths over these opposing gradients to maximize resource acquisition, while avoiding competitive exclusion [5,6,7]. If species can differentiate their niches (light and nutrient requirements in particular) and spatially segregate either through active motility or through differentiated growth rates over the vertical dimension, the amount of spatial overlap (*SO*) within the community should then decrease.

A handful of observational studies have linked species segregation (or its corollary, spatial overlap) to heterogeneous resource distributions [5,8]. Combining field observation and predictive modelling, Clegg et al. [8] observed an increase in flagellate diversity with stratification, which they linked to fine-scale species segregation over opposing resource gradients. In a study across multiple north-temperate lakes, more strongly stratified lakes had reduced *SO* amongst major phytoplankton groups than more mixed lakes and greater taxonomic evenness [9]. Similarly, George and Heaney [10] demonstrated that the physical environment can be one of the main drivers of phytoplankton spatial distribution. High levels of mixing homogenized nutrient concentrations within the water column thereby increased *SO*, based on both the disruption of the nutrient gradient as well as of the active position regulation by phytoplankton.

If the vertical distribution of species in the water column affects competition, the levels of *SO* for a given phytoplankton community should affect its diversity, both taxonomically and functionally. Reduced *SO* should be associated with lower levels of interspecific competition, and consequently higher taxonomic diversity, as more species can coexist in the community (richness) and as spatial differentiation precludes dominance (more evenness). On the other hand, while taxonomic diversity should decline as *SO* increases (more competitive exclusion), the species that persist together should be more functionally diverse in their resource acquisition traits in order to permit some form of segregation along additional niche axes (e.g., resource acquisition rates, storage capacity, trophic strategies) instead of spatially. For example, Stomp et al. [11] demonstrated experimentally that coexistence in a mixed system is possible between spatially overlapping cyanobacterial taxa with different photosynthetic pigment types, i.e., different light requirements. However, greater functional diversity could also be expected under low levels of *SO* when conditions are stratified, given that species growing at different depths face different local conditions that could select for a larger range of trait values. While they did not find any direct relationship between diversity and *SO*, Beisner and Longhi [9] observed increases in phytoplankton taxonomic diversity and functional richness (in motility and resource acquisition traits) for deeper, stratified lakes with clear water columns, where phytoplankton could reduce their *SO*, compared to shallow, polymictic lakes. It is also worth noting that the physical structure of the environment can itself directly influence community composition. For example, Reynolds et al. [12] showed in a mesocosm experiment that altering the mixing depth affected the phytoplankton community composition, with shallower mixing depths favoring sinking diatoms. The composition of the zooplankton grazer community is also likely to affect the diversity of the phytoplankton community through top-down interactions. Currently, we lack a clear understanding of the effect of *SO* on taxonomic or functional trait diversity in natural communities under experimental conditions that control for extraneous factors such as lake morphometry, seasonality and grazer community.

To complement modeling and observational work done to date on the effect of spatial overlap and resource competition on phytoplankton diversity, we conducted an in situ experiment manipulating the water column stratification in a small lake with multiple basins. Our goal is to increase mechanistic understanding of (i) the effect of thermal stratification disruptions of the water column on phytoplankton *SO*, (ii) the effect of *SO*, in conjunction with the physical structure of the water column and top-down interactions, on community taxonomic and functional diversity; all while controlling for lake morphometry, chemistry and global community composition. Data were from a whole-lake experimental thermal stratification manipulation of a temperate lake (Thermocline Induced Mixing Experiment; TIMEX) and used here to assess phytoplankton community diversity in conjunction with overlap (*SO*) between major phytoplankton groups. A previous study by Ouellet Jobin and Beisner [13] showed that the TIMEX treatment application led to thermocline deepening as planned. This deepening of the warmer upper mixed layer (eplimnion) could impede species coexistence (reduced diversity predicted) owing to mixing across greater depth for the same wind. Experimentally deepened thermoclines also resulted in the nutrient gradient being pushed deeper with the hypolimnetic waters, while the light gradient remained unchanged. Ouellet Jobin and Beisner [13] also showed that the treatment application led to some metalimnetic thickening, at the expense of a deeper mixed layer, thereby increasing overall water column stability. This environmental shift should improve the ability of phytoplankton to segregate and avoid competitive exclusion across a more stable water column (increased diversity predicted).

By differentially altering the stratification structure of the different basins of the lake all possessing similar morphometries, we expect to have selectively altered the ability of phytoplankton species to spatially segregate. Within this experimental context, we examined how the spatial overlap (*SO*) of major phytoplankton groups relates to the overall community (taxonomic and functional) diversity, while accounting for other often time-varying changes in the background environmental (thermal stratification) and biotic (zooplankton grazing) variables. Previous results from the TIMEX experiment have shown important shifts in zooplankton composition with thermocline deepening from large-bodied cladocerans to smaller crustacean zooplankton composed of cyclopoid copepods and *Bosmina* spp. [14,15]. Less efficient feeding by these smaller zooplankton associated with deeper thermoclines could also promote phytoplankton diversity. More generally, any change in the zooplankton community might affect phytoplankton diversity through altered top-down effects [16].

**Figure 1:**
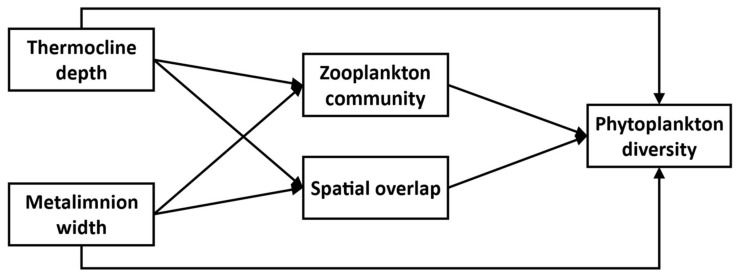
Initial Structural Equation Model. Each box represents a variable, and each arrow is a hypothesized relationship.

Our focal experimental system thus consists of five main interacting compartments: *Thermocline depth, Metalimnion width*, *Zooplankton community*, and *SO*, all potentially influencing the *Phytoplankton diversity* response (Figure 1). We further assume that changes in *Thermocline depth* and *Metalimnion width* through experimental thermocline deepening potentially affects every compartment in the system. We expect *Phytoplankton taxonomic diversity* to decline with greater *SO* because of greater competition between overlapping taxa. On the other hand, increased *SO* could favor greater *Phytoplankton functional diversity* through greater trait variation amongst co-existing taxa, although this effect might be mitigated if, under a lower *SO* regime, species established at different depth experience different environmental conditions, leading to trait differentiation. Finally, we expect changes in the *Zooplankton community* to affect *Phytoplankton diversity* through altered top-down effects and, in particular negative effects on diversity of increasing Cladoceran biomass.

## 2. Materials and Methods

The Thermocline Induced Mixing EXperiment (TIMEX) was conducted from 2007 to 2012 (with 2007 and 2011 being non-experimental years when no treatment was applied) in Croche Lake (45.590 3500 N, 74.000 2800 W) at the Station de biologie des Laurentides, St-Hippolyte, Quebec, Canada. Phytoplankton compositional data was only collected from 2009 to 2011. Samples were collected fortnightly during the day from ice-off to the end of September in each year at a sampling platform anchored at the deepest point of each basin. The main goal of the TIMEX experiment was to alter the depth of the thermocline of one of three lake basins. However, the treatment altered additional parameters related to the stratification structure as well, especially the width of the metalimnion, which was enlarged with thermocline deepening [13].

### 2.1. Experimental Setup

**Figure 2:**
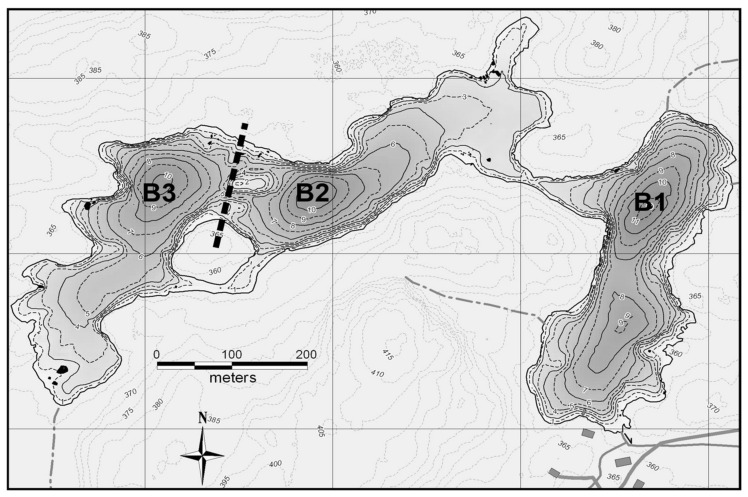
Bathymetric map of the lake (Courtesy of R. Carignan, Station de Biologie des Laurentians, University of Montreal, Montréal, Quebec). The dotted line represents the curtain and each basin is defined as follows: B1 = control, B2 = passively deepened, B3 = actively deepened. Adapted from Ouellet Jobin and Beisner [13].

**Figure 3:**
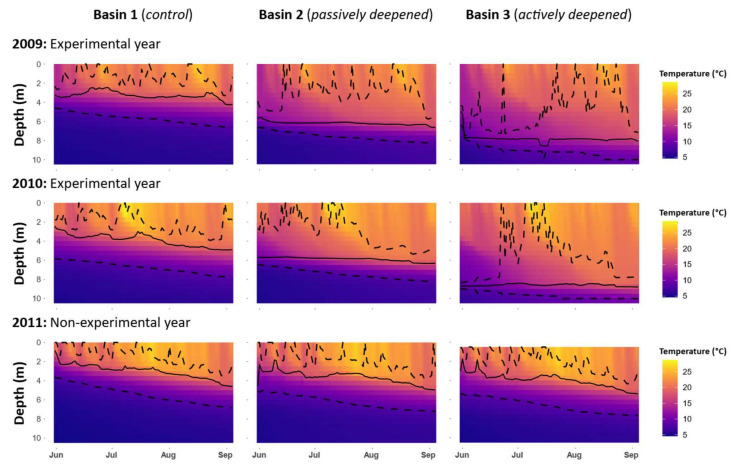
Contour plots of temperature at each depth in 2009, 2010 and 2011 in each of the three basins. The solid line represents the thermocline depth (computed daily), while the dotted lines represent the limits of the metalimnion.

Croche Lake is a meso-oligotrophic and P-limited (TN:TP = 41) as is typical of north temperate lakes with a TN:TP > 21 [17]. The phytoplankton community is mainly dominated throughout the growing season by chrysophytes, diatoms and cryptophytes (Figure S1). The lake is naturally divided into three 10–11-m deep basins (Figure 2). The eastern basin (B1) served as a control in all years. To lower the thermocline in the western-most basin (B3), a solar-powered lake mixer (SolarBee^®^, H_2_O Logics Inc., Sherwood Park, Alberta, Canada) was run during the experimental years (2009–2010 in our dataset). Thermocline depth in the western B3 basin was successfully lowered from 4 m to around 8 m. This basin was isolated from the intermediately located basin (B2) by a narrow pass of 1 m deep water, an island and a 120 m wide and 6 m deep section where a black polyethylene curtain was installed. In all treatment years, passive heat transfer occurred through the curtain, thereby also lowering the thermocline in B2 from 4 m to around 6 m (Figure 3). For simplicity, the basins will be referred to as the control basin (B1), the passively deepened basin (B2) and the actively deepened basin (B3). This setup is described in other related publications [13,14,18].

### 2.2. Data Collection

TP concentrations were measured at the surface and at 2, 4, 6 and 8 m depth in each basin over the three years. A graphical investigation confirmed that the nutrient vertical gradient, present in the control basin B1, was disrupted in B3 during the experimental years (Figure S2).

Whole water column samples were taken for phytoplankton from each basin on each of 22 sampling occasions across the 3 years. An integrated 1.5 cm diameter PVC tube sampler was used to sample from the surface to 1 m above the sediments. Taxa composing the communities were identified and enumerated using the Ütermohl method on an inverted microscope (400× magnification). Biovolumes (in mm^3^.m^−3^) were determined based on measured cell dimensions and by applying geometric formulae for similarly shaped objects [19].

On each sampling date, a FluoroProbe (FP; bbe-Moldaenke, Kiel, Germany) was used to measure the quantity of chlorophyll *a* (Chl *a*) associated with four major spectral groups throughout the water column at the deepest point in each basin. The FP detects Chl *a* by fluorescence using excitation light sources at different wavelengths, which enables grouping of phytoplankton according to their accessory pigments: BROWNS (diatoms, dinoflagellates and chrysophytes), GREENS (chlorophytes), CYANOS (phycocyanin-containing cyanophytes) and MIXED (cryptophytes in this lake). A UV-excitation source is used to subtract the fluorescence coming from chromophoric dissolved organic matter (CDOM). The FluoroProbe data have a vertical resolution of about 10 cm.

Also, at the deepest point in each basin, water temperature profiles were measured at 20 min intervals using thermistor chains equipped with HOBO temperature loggers (±1 °C; Onset Computer Corporation, Cape Cod, MA, USA) at 0.5 m depth intervals and installed for the duration of the experiment. Owing to a defective sensor, temperature data were not available at the surface (0 m) of B3 in 2011. However, this did not affect any subsequent estimations of thermal profile properties in the basin as it was always shallower than the upper limit of the metalimnion.

Finally, the zooplankton community was sampled by hauling a 54 µm mesh net (150 cm in length; 30 cm in diameter) from the bottom of the lake (1 m above the sediments) to the surface at the deep station in each basin. Taxonomic identification was performed on sub-samples using an Olympus inverted microscope (×100 magnification), until a minimum of 200–300 total individuals had been enumerated. Biomasses (dry weight, in µg.L^−1^) were estimated by applying length–mass relationships [20] to standard length measurements of 20 individuals per species. The time series dynamics by basin and year for the cladoceran zooplankton and total phytoplankton (Chl *a*) biomasses are shown in Figure S3. The experimental effect of the TIMEX experiment itself on the interactions of these communities has been explored in detail previously in Gauthier et al. [15] and Sastri et al. [18].

### 2.3. Estimation of Indices and Metrics

Our dataset included 66 sampling events corresponding to unique combinations of sampling date and sites (basins). Overall, there were 21 observations in 2009 (7 time points per basin), 24 observations in 2010 (8 time points per basin) and 21 observations in 2011 (8 time points in B1; 7 in B2; 6 in B3). Index determination and statistical analyses were done in R version 4.1.0 [21]. Indices related to physical structure of the water column (thermocline depth *Thermo_Depth_* and metalimnion width *Meta_Width_*) were estimated for each sampling event from the temperature profiles using the *rLakeAnalyzer* R package [22]. *rLakeAnalyzer* estimates a density gradient over the water column [23]. Thermocline depth is the depth at which this gradient is maximized. The upper and lower bounds of the metalimnion are defined as the depths at which the density gradient reaches a specified threshold value of 0.1 kg.m^−3^.m^−1^.

The FluoroProbe spectral profiles were used to calculate an index of spatial overlap (*SO*) between the spectral groups using a script for overlap in traits created by Mouillot et al. [24] and modified in Beisner and Longhi [9] for phytoplankton profiles. In summary, a kernel density function was applied to the vertical profile of each spectral group taken at a given sampling event. The proportion of the area under the overlapping curves shared between each pair of spectral groups was calculated with the mean of all these pairwise comparisons representing *SO*. The index ranges from 0 to 1, where 0 indicates no spatial overlap and 1 represents total overlap in the distribution curves of the spectral groups.

Phytoplankton taxonomic diversity was estimated using the Shannon diversity index (*H’*) based on biomass with the *vegan* R package [25]. The Shannon index takes into account both the number of species and their relative biomass. A higher Shannon index value indicates a more diverse community, i.e., a community with more species and/or a set of species that contribute more evenly to the total community biomass [26]. Phytoplankton functional diversity was estimated from a functional trait matrix compiled by our research team for lake phytoplankton taxa across lakes in the region (DataS1). This matrix included six functional traits related to morphology and resource acquisition, as listed in Table 1. The traits used have been described in other publications and reflect important processes, in particular resource acquisition [27,28]. All traits were categorical except for the continuous maximum linear dimension (MLD). The MLD is a measure of the size of a cell for a given taxon (in µm). Nitrogen fixation is the potential for some cyanobacteria taxa to fix atmospheric dinitrogen. Silica fixation refers to the production of external protective silica structures by diatoms and by some chrysophytes. Mixotrophy is the potential for a given taxon to acquire energy and nutrients through both phototrophy and phagotrophy via bacterivory. Coloniality refers to the tendency of some taxa to form chains or colonies of multiple cells. The Pigment trait comprises five categories corresponding to combinations of accessory pigments found in one or several taxonomic groups: Brown (diatoms, dinoflagellates, chrysophyceae), Green (chlorophyceae, euglenophyceae), Blue-Green (cyanobacteria), Red (cryptophyceae) and Yellow-Green (xantophyceae). Traits related to nutrient consumption kinetics (e.g., half saturation constants, maximum absorption rates) could not be included in the trait matrix as they are not routinely available for freshwater taxa. However, several studies have noted that these traits correlate well with the body size of phytoplankton [29,30], making MLD a suitable proxy of life history strategy variation. We did not include a trait for motility structures like flagella or gas vacuoles, as motility is likely to directly affect spatial overlap, unlike the traits we chose to consider, that are also more direct proxies of resource competition. Moreover, motility was highly correlated (*ρ* = 0.988) with mixotrophy, and we chose to use the trait most closely related to resource acquisition in this case. The trait values were assigned using microscopic observations and information available in the literature [31]. Phytoplankton functional dispersion (*F_Dis_*) was estimated by applying our trait matrix with the genus biovolume matrix using the *FD* R package [32,33]. *F_Dis_* measures the dispersion of the taxa in the multidimensional space formed by the functional traits and the index increases with greater diversity. It corresponds to the mean distance of individual taxa, weighted by the relative abundances of the taxa, to the community centroid projected in trait space. Furthermore, this index can use both quantitative and qualitative traits and is not sensitive to community taxonomic richness.

A caveat of our study relates to the scale discrepancy in the data used to compute the *SO* and the diversity indices: *SO* was estimated using pigment measurements that combine broad phytoplankton taxonomic groups at fine spatial scales, while diversity was estimated at a finer taxonomic resolution using genus-level biomass, but across the water column. While in theory *SO* could be measured at a finer taxonomic scale by using phytoplankton counts in samples taken at many discrete depths, the sampling and counting effort required would be monumental and is simply not realistic in the context of measurements taken at multiple timepoints as in the TIMEX experiment. For the tools available to us during this unique whole-lake experiment, these discrepancies in scales were unavoidable, but our interpretation considers this context.

### 2.4. Statistical Analyses

All 66 sampling events in our datasets were treated as independent observations. Causal relationships and links between variables were tested using structural equation modeling (SEM). This multivariate statistical framework allows evaluation of the network of causal relationships linking multiple variables based on user-specified hypotheses. The validity of the model is then assessed by confronting it with measured data [34,35]. We first specified a general model that included the ecologically plausible pathways between five compartments: *Thermocline depth*, *Metalimnion width*, *Zooplankton community*, *Spatial overlap*, *Phytoplankton diversity* (Figure 1). The relationships between these compartments reflect the hypotheses of our study. *Thermocline depth* (*Thermo_Depth_*) and *Metalimnion width* (*Meta_Width_*) directly relate to the treatment applied to our system, so we considered these compartments as exogenous variables, not affected by the other variables. All the other variables are endogenous, dependent on at least these two variables. Using this general model (Figure 1) as a template, two distinct models were estimated for each type of community diversity, the first one using *H’* (taxonomic) and the second using *F_Dis_* (functional) to represent the *Phytoplankton diversity* compartment. Variables representing the remaining compartments were identical across the two diversity models. The *Zooplankton community* compartment consisted of Cladoceran biomass (*Cladocera_Biom_*) as these are the most efficient grazers constituting a reliable indicator of the intensity of top-down interactions shaping phytoplankton diversity. Furthermore, a previous analysis of the response of the zooplankton to the TIMEX experiment showed that cladocerans were particularly susceptible to the alteration of stratification structure [15], justifying causal relationships between the compartments *Thermo_Depth_*, *Meta_Width_* and *Cladocera_Biom_* in the SEMs. Because SEMs do not support variables with very dissimilar observed variance, variables were transformed when necessary to stabilize variance. In particular, the variable *Cladocera_Biom_* was log-transformed. We checked for signs of temporal autocorrelation in the variables for each year and for each basin and found none.

Path significance and coefficients were determined through global estimation using the *lavaan* R package [36]. This procedure determines path coefficients by minimizing the difference between the model-implied variance–covariance matrix and the observed data variance–covariance matrix; with the fit between the matrices being assessed by a Chi-square test. A non-significant Chi-square test indicates that the covariance matrices do not differ and that the model structure thus fits the data well. Owing to the non-normal distributions of several variables, we used a bootstrap procedure available in *lavaan* (10,000 bootstrapped samples) to estimate the *p*-values of the model paths. Additional metrics can be used to assess model fit, including the goodness-of-fit index (GFI). This index measures the relative proportion of variance and covariance in the data covariance matrix predicted by the model-implied covariance matrix. A value > 0.95 is indicative of a good fit [37]. For each endogenous variable, an *R^2^* score can be calculated to quantify the amount of variation explained.

We further investigated the effects of environmental factors on individual functional traits. Multiple linear regression analyses were performed on indices of variability for each functional trait used to compute *F_Dis_*, with *Thermo_Depth_*, *Meta_Width_*, *Cladocera_Biom_* and *SO* as predictors. For the quantitative trait, MLD, variability was estimated as the community-weighted standard variance (*CWvar_MLD_*), using a formula proposed by Peres-Neto et al. [38]. Applying the community-weighted variance formula to a quantitative trait yields a weighted measure of the dispersion in trait values within the community. For the six other qualitative traits with two to five different modalities, we estimated the biomass of each trait modality in the community on each sampling date by summing biomasses across taxa presenting that modality. We then applied the formula of the Shannon index (*H’*) on each trait, using the biomasses of the different modalities of a given trait rather than taxa as distinct statistical individuals. Applying the Shannon index formula to a given qualitative trait reflects both number of trait modalities represented in the community, as well as the evenness of the biomass distribution of those modalities. These indices of variability were used as response variables in separate regression models: in total, six regression models were estimated (Table 2). The distributions of the response variables did not always meet the assumptions of frequentist Gaussian models, so we applied permutation tests (10,000 draws) to assess the significatively of the coefficients of the regression models, using the function *lmorigin* available in the R package *ape* [39].

## 3. Results

### 3.1. SEM for the Taxonomic Diversity

**Figure 4:**
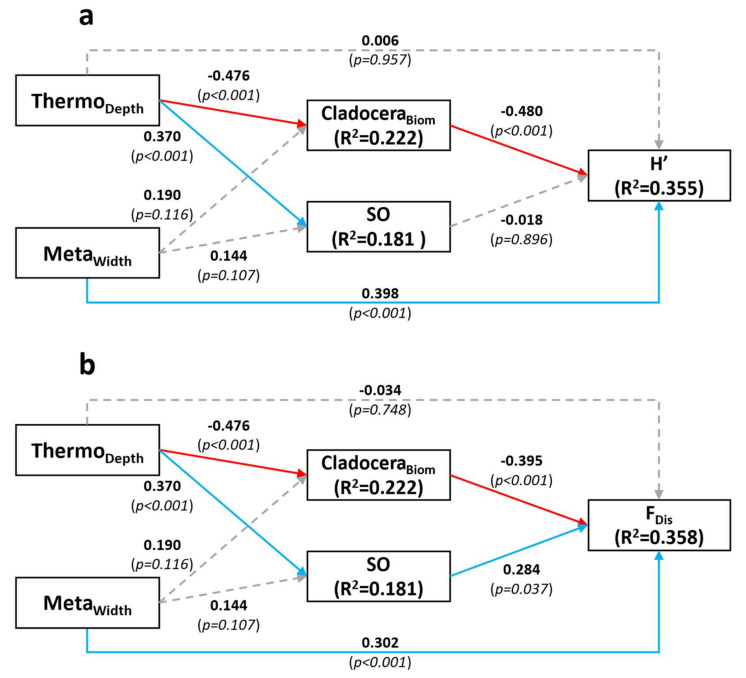
Structural Equation models for *H’* (**a**) and *F_Dis_* (**b**). Dashed grey arrows represent non-significant relationships. Blue arrows represent significant positive relationship and red arrows represent significant negative relationship. Results shown are standardized coefficients and *p*-value (between parentheses), as well as *R^2^* scores for endogenous variables. Abbreviations are as follows: Thermo_Depth_ = thermocline depth, Meta_Width_ = metalimnion width, Cladocera_Biom_ = cladoceran biomass, SO = spatial overlap, H’ = Shannon diversity index, F_Dis_ = Functional dispersion.

The SE model for taxonomic diversity H’ was not significant ($\chi^{2}=0.404,\;df=1,\;p=0.525$) and the goodness-of-fit index was high (GFI=1), together indicating a valid model adequately representing the observed data (Figure 4a). The relationship of *H’* with *SO* was not significant and neither was the direct relationship with *Thermo_Depth_*. However, taxonomic *Phytoplankton diversity* (*H’*) was significantly positively affected by *Meta_Width_*. *H’* was also significantly negatively affected by *Cladocera_Biom_* (*Zooplankton community* compartment), which itself was negatively affected by *Thermo_Depth_* (but not by *Meta_Width_*). A significant positive relationship was detected between *Thermo_Depth_* and *SO*, but not between *Meta_Width_* and *SO*. Although there was no direct significant relationship between *Thermo_Depth_* and *H’*, a larger epilimnion indirectly promoted the taxonomic *Phytoplankton diversity* compartment because *Thermo_Depth_* negatively affected *Cladocera_Biom_*, which itself negatively affected *H’*. The model explained 18.1% of the variability in *SO*, 22.2% of the variability in *Cladocera_Biom_* and 35.5% of the variability in *H’*.

### 3.2. SEM for the Functional Diversity

The final SE model for *F_Dis_* was very similar to the model for *H’* (Figure 4b), as might be expected given that the predictors for the compartments were the same. The main difference was that this SEM revealed a significant positive effect of *SO* on *F_Dis_*, indicating that increased spatial overlap favored functional *Phytoplankton diversity*. The model Chi-square test was not significant ($\chi^{2}=0.405,\;df=1,\;p=0.525$) and the goodness-of-fit index was GFI=1, indicating that the model provided an adequate fit to the data. This model explained 18.1%, 22.2% and 35.8% of the variability of *SO*, *Cladocera_Biom_* and *F_Dis_*, respectively.

### 3.3. Effect of SEM Predictors on the Diversity of Individual Traits

We used *Thermo_Depth_*, *Meta_Width_*, *Cladocera_Biom_* and *SO* as predictors in the permutation multiple linear regression models on the individual trait variability indices (Table 2). *Thermo_Depth_* had a significant negative effect on the diversity of taxa cell sizes (*CWvar_MLD_*) and a significant positive effect on the diversity of pigments (*H_Pig_*). *Meta_Width_* significantly and positively affected the diversity of the nitrogen fixation, mixotrophy and pigment traits (*H_Fix_*, *H_Mix_* and *H_Pig_* respectively). *Cladocera_Biom_* had a significant negative effect on diversity of the pigment trait (*H_Pig_*) and the coloniality trait (*H_Col_*). *SO* did not significantly affect any individual trait diversity indices, although all regression coefficients in relation to *SO* were positive.

## 4. Discussion

We examined, in a whole-lake experimental context, whether altering the stratification structure of the water column would reveal an influence of spatial overlap on community diversity. Only functional diversity (*F_Dis_*) was directly influenced by spatial overlap: higher *SO* was associated with a greater diversity of the resource acquisition and morphology traits we considered in our analysis. While we anticipated that a positive effect of *SO* on functional diversity could be attenuated by enhanced functional differentiation at low levels of *SO* as spatially segregated taxa need to adapt to different local conditions, the results obtained were in line with our theoretical expectations that, to coexist, spatially overlapping species need to be functionally distinct, notably in their resource requirements or feeding strategies. However, we also predicted that increased interspecific competition would reduce taxonomic diversity, but our analyses revealed no such effect of *SO* on the Shannon diversity index (*H’*) of the community. These contrasting diversity results do make sense however if the functional trait differentiation is effective at precluding taxonomic diversity decline through niche partitioning.

In our study, greater *SO* was associated with a deeper thermocline (Figure 4a,b), and thus, by definition, a wider mixed (epilimnetic) layer. Overall, this implies a larger portion of the water column over which phytoplankton species cannot easily regulate their position and are thus potentially susceptible to greater competition. Therefore, we expected to see a negative effect of *SO* on *H’*. The absence of such signal indicates that the effect of spatial aggregation on diversity might not be as straightforward as we initially assumed, and that species can coexist even when spatial overlap is high—perhaps via coexistence of taxa utilizing different traits. The absence of effect of *SO* on *H’* could also simply indicate that interspecific competition is not a strong driver of taxonomic diversity, owing to increased functional diversity or trait variation. Furthermore, the physical structure of the environment and top-down interactions also appear to be important drivers of diversity, as *Meta_Width_* and *Cladocera_Biom_* significantly affected *H’* and *F_Dis_* in our SE model (Figure 4a).

The SEMs featured a direct positive effect of metalimnetic width on both diversity types, but not a direct effect of thermocline depth itself. Focusing on functional diversity, further analyses revealed an effect of metalimnetic width on the diversity of pigments and trophic strategy traits (mixotrophy and diazotrophy). A wider metalimnion implies a thicker stable layer covering a larger range of light intensities and colors. Species with different light requirements, hence with different pigment types, would be able to better coexist within a wider stratified layer by establishing at different depths [40]. The positive effect of *Meta_Width_* on *H_Mix_* and *H_Nfix_* appears to mostly be the result of a taxonomic change in community contribution. Further investigation revealed that most of the biomass is mixotrophic when the metalimnion is thin, and that the prevalence of mixotrophy in the community is negatively affected by a thicker *Meta_Width_* (Figure S4a). On the other hand, a larger metalimnion, implying a larger stratified portion of the water column, would favor buoyant cyanobacteria that can use gas vacuoles to regulate their vertical positions (Figure S4b) [41,42]. A higher contribution of autotrophic cyanobacteria would reduce the prevalence of mixotrophy, thus promoting a better balance between the prevalence of autotrophic and mixotrophic taxa. Since some cyanobacterial taxa are able to fix dinitrogen, a larger metalimnion would then also contribute to a diversification (*H_Nfix_*) of nitrogen fixation strategy [43].

While thermocline depth did not have a similar direct significant effect on the overall functional diversity of the community, it did affect the diversity of several individual traits. In particular, a deeper thermocline positively affected the diversity of community cell sizes and negatively affected the diversity of pigments (*CWvar_MLD_* and *H_Pig_*, respectively). A larger mixed layer induced by thermocline deepening might allow larger sinking diatoms to be more prevalent where otherwise small non-sinking taxa would dominate. Indeed, Ptanick et al. [44] demonstrated in a mesocosm experiment that large fast-sinking diatoms benefit from higher mixing depths. Conversely, a deeper epilimnion could prevent some species from establishing at the optimal light absorption depth for their accessory pigment composition, leading to a loss of pigment diversity in the community; optimal adaptations being for varying light (more mixed taxa) or for reduced light (those that are able to remain near or in the hypolimnion). These effects of thermocline depth and metalimnetic width on phytoplankton diversity illustrate how the physical environment shapes community composition.

Returning to the significant relationship between *SO* and *F_Dis_*, we expected higher levels of *SO* to be associated with higher levels of functional differentiation. When spatial niche overlap occurs within the actively mixed layer, species need to display different strategies of nutrient acquisition to avoid competitive exclusion [45,46]. More generally, functional differentiation of traits related to resource acquisition should promote coexistence. However, we found no positive significant effect of *SO* on any individual trait diversities. We noted a near-significant trend (*p = 0.060*) indicating that *SO* might promote a better balance between silica-requiring taxa (i.e., diatoms and chrysophytes) and non-silica-requiring taxa (greater *H_Si_*). Overall, our results indicate that *SO* acts on the global functional diversity of the community by affecting the combination of multiple interacting traits, which is captured by a global index of trait diversity like *F_Dis_*, rather than on the diversity of individual trait types. Note that these results are conditioned by the selection of traits we could measure and chose to include in our analyses.

Grazing by zooplankton was also an important factor in regulating phytoplankton diversity in our SEM analyses. In particular, cladoceran biomass was one of the main factors affecting, negatively, phytoplankton, both taxonomic and functional diversity. The *Cladocera_Biom_* effect on *F_Dis_* was greater than *SO* in terms of the absolute values of the standardized relationship coefficients, indicating that the zooplankton community was a more important driver of *Functional diversity* than was *SO* in the context of our experiment. The negative grazing effect runs counter to theory that states that zooplankton grazing pressure should promote phytoplankton taxonomic diversity by reducing the amount of interspecific resource competition [47,48], even experimentally for evenness [49]. However, detailed examination of phytoplankton communities under increasing levels of cladoceran grazing has demonstrated concomitant shifts to dominance by larger or colonial phytoplankton species [50], thereby reducing functional diversity, and thus potentially taxonomic diversity where such species are rare, as is the case in our study lake and as we observed. Indeed, individual trait diversity did demonstrate significantly reduced diversity within traits associated with coloniality (*H_Col_*) and pigments (*H_Pig_*), indicating that selective grazing by cladocerans can reduce the diversity of certain phytoplankton trait types as a result. Accompanying declines in taxonomic diversity would be expected in a relatively closed experimental system such as ours where selective feeding could remove entire taxa (based on traits) without replacement by other more resistant species from adjacent lakes (none upstream of our site) over the time scale of our experiment. Indeed, cladoceran feeding is known to be selective, as observed in experiments demonstrating that cladoceran gut pigment composition is significantly different from the pigment composition of the associated phytoplankton community [51].

It is important to note that our spectral measurements of phytoplankton vertical structure can only approximate real values of *SO*, as they only inform on the pigment levels for four broad spectral groups, but at fine spatial scales. For example, we cannot quantify spatial overlap between chlorophyte taxa, as they all share the same green pigment detected spectrally. This leads to difficulty in fully assessing *SO* at very fine taxonomic scales, similar to those at which diversity was estimated. To utilize whole-lake experiments to their full potential, improved rapid tools to assess both spatial overlap at fine spatial scales and taxonomic resolution are needed.

## 5. Conclusions

Our study revealed that altering the thermal stratification structure of a lake, while controlling for lake morphometry, chemistry and global community composition, can affect spatial overlap amongst phytoplankton groups. Spatial overlap was related to greater functional diversity, indicating that forced coexistence enabled niche differentiation along trait axes to alleviate interspecific competition, that appear to have precluded an effect of *SO* on taxonomic diversity. Globally, however, our analyses revealed that the physical structure of the environment and cascading top-down interactions are likely the stronger drivers of phytoplankton diversity (both taxonomic and functional). To our knowledge, this study is the first to simultaneously assess the relative effects of not only spatial overlap, but also grazing and the physical environment on multiple dimensions of phytoplankton diversity.

## Figures and Tables

**Table 1:** List of the functional traits used in the study, their type and diversity index to which they are associated.

Functional Traits	Values	Associated Diversity Index
Maximum Linear Dimension (MLD)	Quantitative (µm)	*CWvar_MLD_*
Nitrogen fixation	Y/N	*H_Nfix_*
Silicium fixation	Y/N	*H_Si_*
Mixotrophy	Y/N	*H_Mix_*
Coloniality	Y/N	*H_Col_*
Pigment	Brown Green Blue-Green Yellow Red	*H_Pig_*

**Table 2:** Results of the permuted multiple linear regressions on the different trait diversity indices. For each regression, the coefficients for each potential explanatory factor (from left to right: *Thermocline depth*, *Metalimnion width*, *Zooplankton biomass* and *Spatial Overlap*) are indicated along with associated *p*-values in parentheses. Significant coefficients and *p*-values are indicated in bold.

	*Thermo_Depth_*	*Meta_Width_*	*Cladocera_Biom_*	*SO*
*CWvar_MLD_*	**156** **(*1.00 × 10^−4^*)**	−16.7 (*0.342*)	3.10 (*0.082*)	85.7 (*0.429*)
*H_Nfix_*	−8.93 **× 10^−4^** (*0.401*)	**8.15 *×* 10^−3^** **(*0.025*)**	2.40 **× 10^−4^** (*0.114*)	0.01 (*0.387*)
*H_Si_*	3.76 **× 10^−3^** (*0.322*)	−4.19 ***×* 10^−4^** (*0.470*)	−4.63 ***×* 10^−4^** (*0.183*)	0.18 (*0.060*)
*H_Mix_*	3.40 **× 10^−3^** (*0.302*)	**0.019** **(*0.004*)**	−5.21 ***×* 10^−4^** (*0.099*)	0.07 (*0.221*)
*H_Col_*	−1.16 **× 10^−3^** (*0.427*)	0.0104 (*0.091*)	**−1.39 *×* 10^−3^** **(*0.002*)**	0.09 (*0.167*)
*H_Pig_*	**−0.0302** **(*0.041*)**	**0.0608** **(*0.001*)**	**−2.61 *×* 10^−3^** **(*0.007*)**	0.26 (*0.121*)

## Data Availability

The datasets and R scripts necessary to reproduce the statistical results presented in this study are available as Supplementary Material (DataS1).

## Supplementary Materials

The following are available online at https://www.mdpi.com/article/10.3390/microorganisms9122447/s1, Figure S1: Time series of the phytoplankton community composition in the TIMEX experiment; Figure S2: Vertical profiles of total phosphorus (TP) in the TIMEX experiment; Figure S3: Time series dynamics for the cladoceran zooplankton and total phytoplankton biomasses (Chl *a*) in the TIMEX experiment; Figure S4: Bootstrapped linear regressions of the metalimnion width over the prevalence of mixotrophy and cyanobacteria; DataS1: Datasets and R scripts necessary to reproduce the SEM results presented in Figure 4, the permutated multivariate regression analyses on individual trait diversity presented in Table 2 and the bootstrapped linear regression models presented in Figure S4.
