# Supplementary material for: Effects of Vertical Spatial Overlap on Phytoplankton Diversity under Experimentally Altered Lake Stratification Regimes
Source: Microorganisms. 2021 Nov 27;9(12):2447. doi: 10.3390/microorganisms9122447 (PMC8708865; doi:10.3390/microorganisms9122447)
Supplement: Supplementary file 1 [file microorganisms-09-02447-s001.zip › SupplementaryMaterials_DataS1_LeNoachetal/ReadmeDataS1_LeNoachetal.pdf]

Supplementary Material for:

Effects of vertical spatial overlap on phytoplankton diversity under experimentally altered lake stratification regimes

Philippe Le Noac'h* (ORCID: 0000-0001-8866-751X)
Vincent Ouellet Jobin
Beatrix E. Beisner (ORCID: 0000-0001-6972-6887)

Department of Biological Sciences, University of Québec at Montréal and Interuniversity Research Group in Limnology/Groupe de Recherche Interuniversitaire en Limnologie (GRIL), C.P. 8888 Succ. Centre-Ville, Montréal, Québec, Canada

Corresponding author: le_noac_h.philippe@courrier.uqam.ca

Readme file S1: Description of the content of *DataS1*

This directory contains the necessary elements to reproduce the results presented in Figure 4 (SEMs), the results presented in Table 2 (permutated multivariate linear regressions on functional trait diversity) and the results shown in Figure S4 (bootstrapped linear regression models of metalimnetic width over the prevalence of mixotrophy and the prevalence of cyanobacteria):

- The dataset *Data_SEM_LeNoachetal.csv*, which contains the variables used in the different statistical analyses: *Thermocline depth* (column *Thermo_Depth*, measured in m), *Metalimnion width* (column *Meta_Width*, measured in m), *Cladoceran biomass* (column *Cladocera_Biom*, measured in $\mu g.L^{-1}$), *Spatial overlap* (column *SO*, dimensionless), *Shannon diversity index* (column *Shannon_H*, dimensionless) and *Functional dispersion* (column *FDis*, dimensionless). The *Date* and *Basin* columns identify each sampling event.

- The dataset *Data_PhytoBiomasses_LeNoachetal.csv*, which contains the biovolume measurements (in $mm^3.m^{-3}$) of the phytoplankton taxa. Each column corresponds to a taxon (except for the *ID* column) and each row to a sampling event. The *ID* column identifies each sampling events with values with the format *[Date]_[Basin]*.

- The dataset *Data_FunctionalTraits_LeNoachetal.csv*, which contains the functional trait values by the phytoplankton taxon (cf. Table 1 of the article). Each column corresponds to a trait (except for the *Taxa, Taxa_abr* and *Groupe* columns) and each row corresponds to a taxon. The *Taxa* column gives the complete genus of each taxon. The values of the *Taxa_abr* are the abbreviated version of the *Taxa* column and correspond to the column names of the *Data_PhytoBiomasses_TIMEX.csv* dataset. The *Groupe* column gives the taxonomic group to which each taxon belongs. All traits are categorical except for the Maximum Linear Dimension (MLD) measured in µm.

- The R script *Script_SEM_LeNoachetal.R*, which contains the code necessary to estimate the bootstrapped SEMs presented in Figure 4. The script uses as input, the dataset *Data_SEM_LeNoachetal.csv*, and returns as outputs (for *nboot* = 10000), the following:
    - *Res_SEM_[Export_id]_TableStandardizedSolutionBoot_BSnboot=10000.csv* (dataset of the bootstrapped p-values of the standardized coefficients values)
    - *Res_SEM_[Export_id]_TableRsq_BSnboot=10000.csv* (dataset of the R^2 values of the endogenous variables)
    - *Res_SEM_[Export_id]_TableFitMeasures_BSnboot=10000.csv* (dataset of the model fit metrics).

  The script can take several minutes to run when the number of bootstrapped samples *nboot* is high.

- The R script *Script_PermutationsMultReg_LeNoachetal.R*, which contains the code necessary to compute the individual diversity indices for each functional trait and subsequently estimate the permutated multivariate linear regression models using the individual diversity indices as response variables. This script uses as input the datasets *Data_SEM_LeNoachetal.csv*, *Data_PhytoBiomasses_LeNoachetal.csv* and *Data_FunctionalTraits_LeNoachetal.csv* and returns as output *Res_MultivlmpermTraits_nperm=10000.csv*, a dataset containing the coefficient values and the corresponding p-values of the different regression models.

- The R script *Script_lmbootMetaWidthCyanoMixotr_LeNoachetal.R*, which contains the code necessary to estimate and plot the bootstrapped linear regression models of metalimnetic width on the prevalence of mixotrophy and on the prevalence of cyanobacteria. This script uses as input, the datasets *Data_SEM_LeNoachetal.csv*, *Data_PhytoBiomasses_LeNoachetal.csv* and *Data_FunctionalTraits_LeNoachetal.csv*, and returns as output *FigureS4a_MetaWidthMixotrophy.svg* and *FigureS4b_MetaWidthCyanobacteria.svg*, the plots presented in Figure S4.
