# Supplementary material for: Effects of Vertical Spatial Overlap on Phytoplankton Diversity under Experimentally Altered Lake Stratification Regimes
Source: Microorganisms. 2021 Nov 27;9(12):2447. doi: 10.3390/microorganisms9122447 (PMC8708865; doi:10.3390/microorganisms9122447)
Supplement: Supplementary file 1 [file microorganisms-09-02447-s001.zip › SupplementaryFigures_LeNoachetal.pdf]

Supplementary Material for:

Effects of vertical spatial overlap on phytoplankton diversity under experimentally altered lake stratification regimes

Philippe Le Noac'h (ORCID: 0000-0001-8866-751X)*
Vincent Ouellet Jobin
Beatrix E. Beisner (ORCID: 0000-0001-6972-6887)

Department of Biological Sciences, University of Québec at Montréal and Interuniversity Research Group in Limnology/Groupe de Recherche Interuniversitaire en Limnologie (GRIL), C.P. 8888 Succ. Centre-Ville, Montréal, Québec, Canada

* Corresponding author: le_noac_h.philippe@courrier.uqam.ca

Figure S1: Time series of the phytoplankton community composition in the TIMEX experiment.

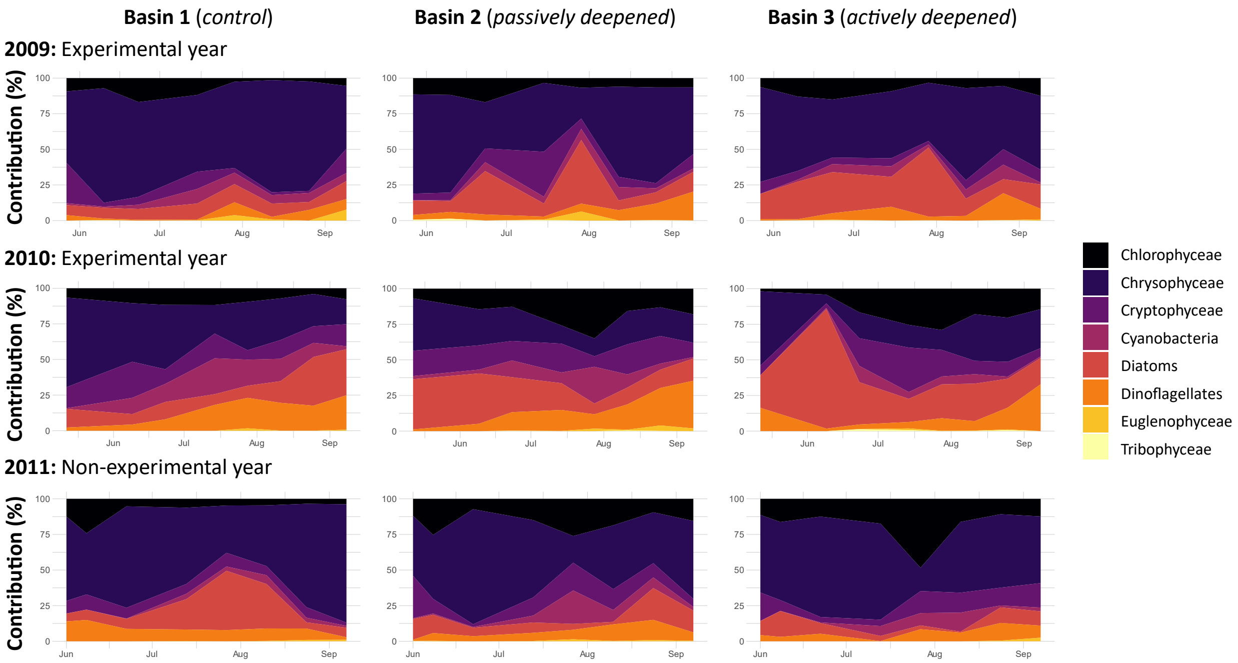

Figure S1. Time series of the phytoplankton community composition in the TIMEX experiment per basin and per year, as a percentage of the contribution of the main taxonomic groups to the total phytoplankton community biomass. Summing all contributions at a given sampling event yields a total contribution of 100%.

Figure S2: Vertical profiles of total phosphorus (TP) in the TIMEX experiment.

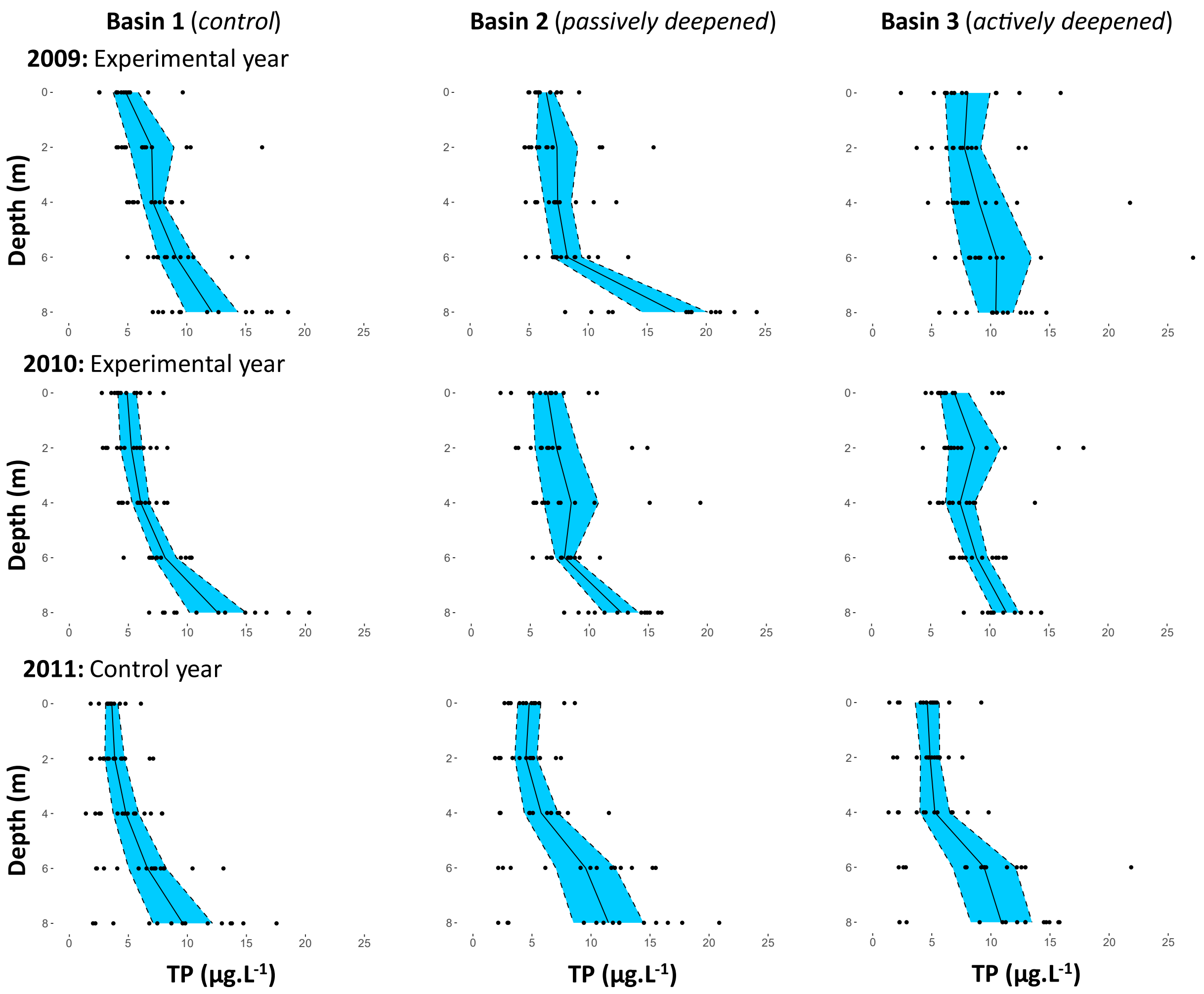

Figure S2. Vertical profiles of total phosphorus (TP) concentrations in 2009, 2010 and 2011 in each of the three lake basins. The black dots are the individual TP concentration measurements. The solid line is the mean of the individual TP concentration over depth. The dotted lines are the limits of the 95% confidence interval around the value mean TP concentration over depth.

Figure S3: Time series dynamics for the cladoceran zooplankton and total phytoplankton biomasses (Chl *a*) in the TIMEX experiment.

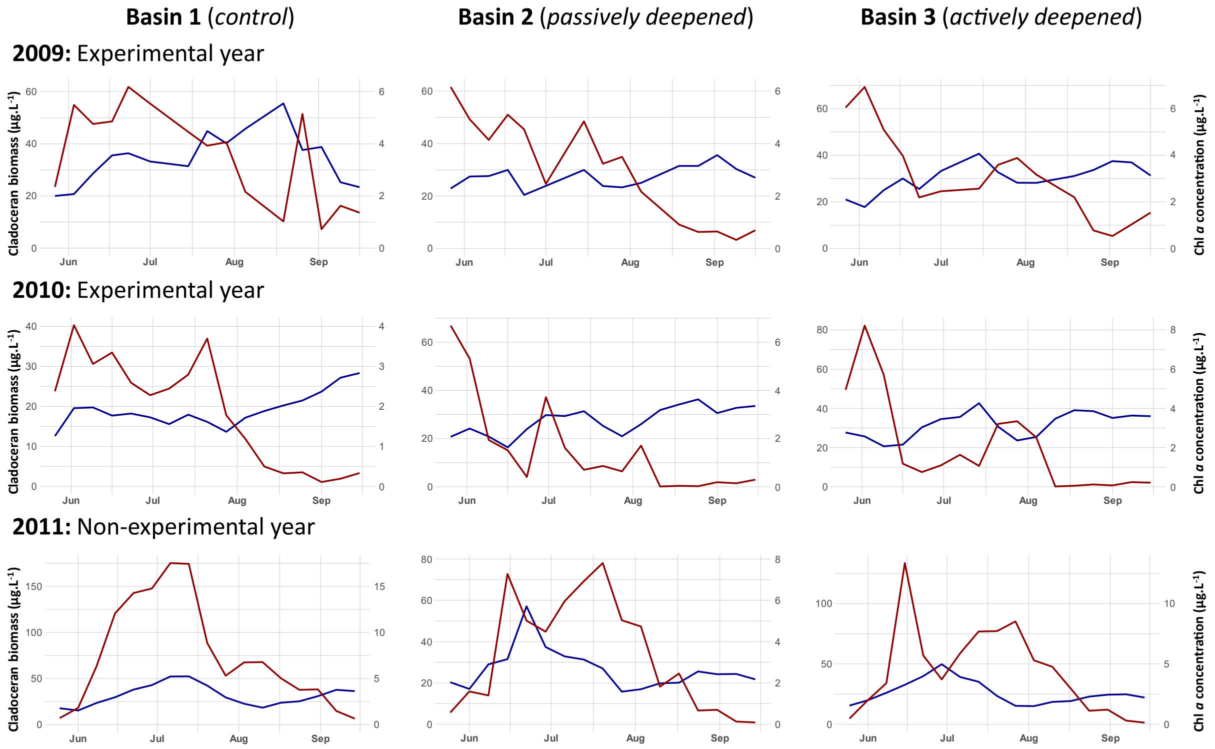

Figure S3. Time series dynamics for the cladoceran zooplankton (red lines) and total phytoplankton (Chl *a*) biomasses (blue lines) by basin and year in the TIMEX experiment. The phytoplankton dynamics were estimated as the mean of the vertical Chl *a* profiles measured by the fluoroprobe over the photic zone (0-7 meters). The values of the cladoceran zooplankton biomasses should be read on the left axes and the Chl *a* concentrations should be read on the right axes.

Figure S4: Bootstrapped linear regressions of the metalimnion width over the prevalence of mixotrophy and cyanobacteria

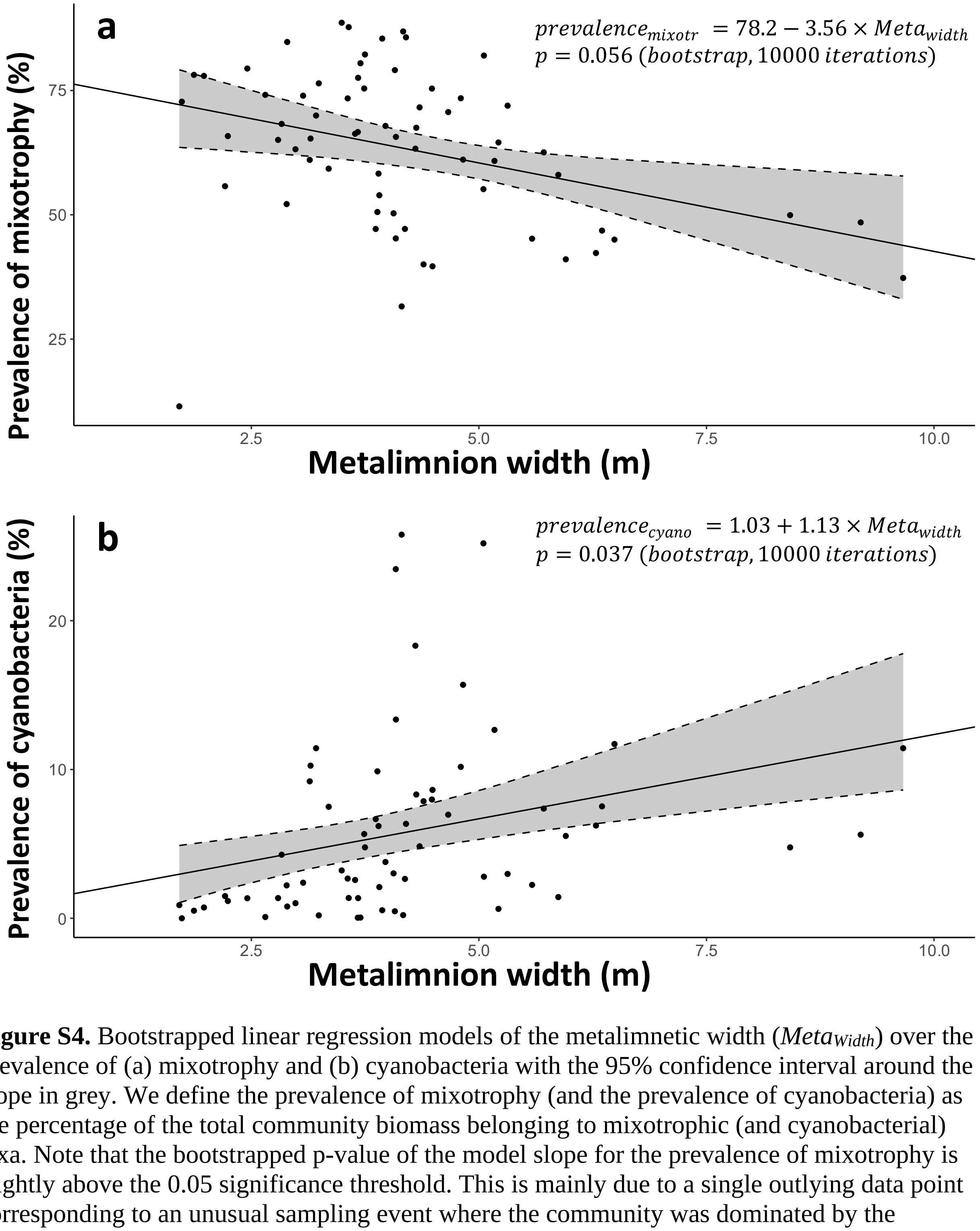

Figure S4. Bootstrapped linear regression models of the metalimnetic width ($Meta_{Width}$) over the prevalence of (a) mixotrophy and (b) cyanobacteria with the 95% confidence interval around the slope in grey. We define the prevalence of mixotrophy (and the prevalence of cyanobacteria) as the percentage of the total community biomass belonging to mixotrophic (and cyanobacterial) taxa. Note that the bootstrapped p-value of the model slope for the prevalence of mixotrophy is slightly above the 0.05 significance threshold. This is mainly due to a single outlying data point corresponding to an unusual sampling event where the community was dominated by the (autotrophic) diatoms *Aulacoseira sp.* and *Asterionella sp.*, with those two taxa contributing to almost 70% of the biomass of the community at this event. When this data point is removed from

the dataset, the bootstrapped p-value of the slope becomes very significant (*p=0.0013*). The bootstrapping procedures were performed using functions from the R package *boot* [1].

Reference

1. Canty, A.; Ripley, B. Boot: Bootstrap R (S-Plus) Functions (Version 1.3.28). **2021**.
